# The Anticancer Effects of FDI-6, a FOXM1 Inhibitor, on Triple Negative Breast Cancer

**DOI:** 10.3390/ijms22136685

**Published:** 2021-06-22

**Authors:** Karan Ulhaka, Kanyanatt Kanokwiroon, Mattaka Khongkow, Rassanee Bissanum, Thanaporn Khunpitak, Pasarat Khongkow

**Affiliations:** 1 Institute of Biomedical Engineering, Faculty of Medicine, Prince of Songkla University, Songkhla 90110, Thailand; k_ulhaka@outlook.co.th (K.U.); ploy_khunpitak@hotmail.com (T.K.); 2 Department of Biomedical Sciences and Biomedical Engineering, Faculty of Medicine, Prince of Songkla University, Songkhla 90110, Thailand; kkanyana@gmail.com (K.K.); rassanee.b@gmail.com (R.B.); 3 National Nanotechnology Centre (NANOTEC), National Science and Technology Development Agency, Pathumthani 12120, Thailand; mattaka@nanotec.or.th; 4 Translational Medicine Research Center, Faculty of Medicine, Prince of Songkla University, Songkhla 90110, Thailand

**Keywords:** triple negative breast cancer, FOXM1, FDI-6, anti-cancer effects

## Abstract

Triple-negative breast cancer (TNBC) presents an important clinical challenge, as it does not respond to endocrine therapies or other available targeting agents. FOXM1, an oncogenic transcriptional factor, has reported to be upregulated and associated with poor clinical outcomes in TNBC patients. In this study, we investigated the anti-cancer effects of FDI-6, a FOXM1 inhibitor, as well as its molecular mechanisms, in TNBC cells. Two TNBC cell lines, MDA-MB-231 and HS578T, were used in this study. The anti-cancer activities of FDI-6 were evaluated using various 2D cell culture assays, including Sulforhodamine B (SRB), wound healing, and transwell invasion assays together with 3D spheroid assays, mimicking real tumour structural properties. After treatment with FDI-6, the TNBC cells displayed a significant inhibition in cell proliferation, migration, and invasion. Increased apoptosis was also observed in the treated cells. In addition, we found that FDI-6 lead to the downregulation of FOXM1 and its key oncogenic targets, including CyclinB1, Snail, and Slug. Interestingly, we also found that the FDI-6/Doxorubicin combination significantly enhanced the cytotoxicity and apoptotic properties, suggesting that FDI-6 might improve chemotherapy treatment efficacy and reduce unwanted side effects. Altogether, FDI-6 exhibited promising anti-tumour activities and could be developed as a newly effective treatment for TNBC.

## 1. Introduction

Triple negative breast cancer (TNBC) is a type of breast cancer found in 15% of total breast cancer patients [1]. Its important aspect is the absence of three identified main receptors, namely: estrogen receptor (ER), progesterone receptor (PR), and human epidermal growth factor receptor 2 (HER2). As TNBC is heterogenous in nature and lacks the usual molecular targets, treatments for TNBC are far more complicated than for other breast cancer types. Currently, chemotherapy is the standard treatment for TNBC. However, the efficacy is limited and often results in poor outcomes for patient who cannot tolerated the adverse effects or who do not respond well to the usual treatment. Targeted therapies in TNBC are still in the developing process [2]. Therefore, it is not available for use in general practices yet.

There are several studies that have focused on identifying potential targets of TNBC in the last decade. One of the identified targets is Forkhead box protein M1 (FOXM1), a transcription factor in the Forkhead superfamily [2]. FOXM1 normally regulates cell proliferation and cell cycle progression [3,4]. However, FOXM1 overexpression in cancer promotes several aspects of cancer progression [5,6]. FOXM1 is upregulated in several solid tumours, including breast cancer [7,8], and is also correlated with a poor outcome of patients [9,10,11]. Regarding TNBC, the FOXM1 expression was discovered to be higher when compared with other breast cancer types [12,13,14]. The suppression of the FOXM1 expression in TNBC resulted in a decrease in the expressions of the FOXM1 associated pathways, the inhibition of cell proliferation, and in migration. It also induced apoptotic cell death [14,15].

Considering FOXM1 as a potential target for cancer treatment, Gormally et al. discovered that FDI-6 (NCGC00099374) can bind directly to FOXM1, preventing FOXM1 from binding to its genomic target in cancer cells. It induced the downregulation of FOXM1 downstream target genes in cancer cells with a high FOXM1 expression. Furthermore, the FDI-6 specificity has been verified. The effect of FDI-6 was found to be limited to FOXM1 and its downstream targets. Other Forkhead proteins, FOXA1, FOXA2, and FOXP2, which have similar DNA-binding domain to FOXM1, were unaffected by FDI-6, suggesting that FDI-6 is a potential specific FOXM1 inhibitor [16,17].

As FOXM1 was upregulated in TNBC, FDI-6 would be a possible TNBC targeted therapeutic candidate. Therefore, to provide further evidence to support FDI-6 as a viable targeted therapy for TNBC, we aimed to investigate the anti-cancer effects of FDI-6 on tumour cell growth, invasion, and migration, as well as the molecular mechanism involved in TNBC cells using 2D and 3D cell culture models. In the present study, we found that TNBC cells treated with FDI-6 displayed a significant decrease in cell proliferation, migration, and invasion, and an increase in apoptosis. We also investigated the molecular mechanisms and discovered that FOXM1, along with its oncogenic key targets, Cyclin B1, Slug, and Snail, were downregulated after FDI-6 treatment. The reduced expression of Slug and Snail suggested that FDI-6 could suppressed the epithelial mesenchymal transition processes. We concluded that FDI-6 could be a promising candidate for TNBC treatment.

## 2. Results

### 2.1. FDI-6 Inhibits TNBC Cell Proliferation

**Figure 1:**
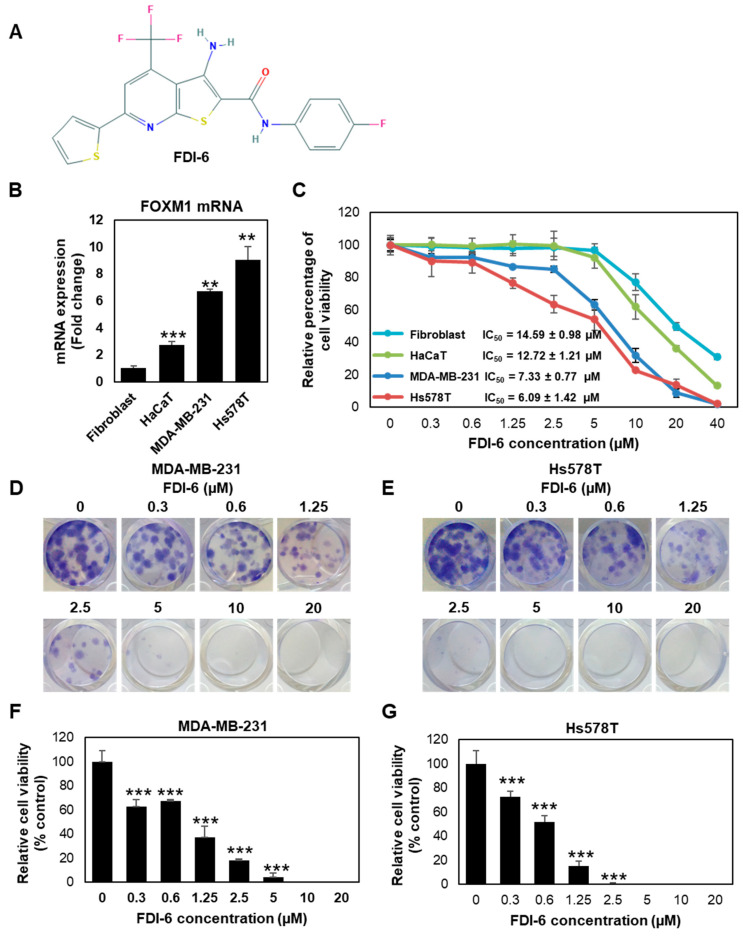
FDI-6 suppressed the proliferation of breast cancer. (**A**) Chemical structure of FDI-6 [18]; (**B**) mRNA expression level of FOXM1 in the cell lines used in this study determined by qRT-PCR after normalizing against L19 the house keeping gene; (**C**) SRB assay results of MDA-MB-231, Hs578T, fibroblasts, and HaCaT; (**D**,**E**) Colony formation images of (**D**) MDA-MB-231 and (**E**) Hs578T cells after being treated with different concentrations of FDI-6 for 72 h, and then incubated for 14 days; (**F**,**G**) Relative cell viability from the clonogenic assay of (**F**) MDA-MB-231 and (**G**) Hs578T cells in comparison with the control group. All bar graphs represent an average of three independent experiments ± S.D. (*n* = 3). Statistical significance was determined by Student’s *t*-test (not significant, ns; significant, ** *p* < 0.01, *** *p* < 0.001).

FDI-6 was recently identified as a small compound with potent inhibitory activities against FOXM1 (Figure 1A). First, we investigated the molecular characterisation of the four cell lines used in this study, consisting of two TNBC cell lines, including MDA-MB-231 and Hs578T, as well as human dermal fibroblasts (HDFs) and keratinocytes (HaCaT) as the non-tumour controls. We found that Hs578T exhibited the highest FOXM1 expression (nine times relative to fibroblasts), followed by MDA-MB-231 (6.7 times relative to fibroblasts), HaCaT cells, and fibroblasts, respectively (Figure 1B). These results show that TNBC cells exhibited a higher FOXM1 expression compared with non-cancer cells, suggesting that these four cell lines could be good models for further anti-cancer activity studies. We then investigated the anti-proliferative effect of FDI-6 using a Sulforhodamine B (SRB) assay. The results demonstrated that FDI-6 inhibits cell proliferation in a dose-dependent manner. After 72 h post-treatment, we found that TNBC, MDA-MB-231, and Hs578T cells were more susceptible to the tested compound compared with the non-tumour fibroblasts and HaCaT cells, as indicated by their half maximal inhibitory concentration (IC_50_) of 7.33 ± 0.77 µM, 6.09 ± 1.42 µM, 14.59 ± 0.98 µM, and 12.71 ± 1.21 µM, respectively (Figure 1C). Interestingly, among the four cell lines, Hs578T cells with the highest FOXM1 expression represented the most responsiveness to the compound, suggesting that FDI-6 inhibited cell proliferation in a FOXM1 expression-dependent manner. Considering more than half of both TNBC cell lines were effectively suppressed at a concentration of 10 µM during the 72-h duration, 61.9% of HaCaT cells and 80% of fibroblasts, representing non-tumour cells, remained viable. We then decided to use 10 µM as an appropriate concentration for further experiments. Additionally, besides triple negative breast cancer cells, FDI-6 also effectively suppressed the growth of FOXM1 expressing ER-positive MCF-7 cells in a dose dependent manner, and the IC50 was 3.227 ± 0.5.3 µM (Figure S1). Next, we evaluated the long-term anti-proliferative effect of FDI-6 by performing a clonogenic assay. Briefly, TNBC cells were treated with FDI-6 for 72 h, then the culture media were replaced and they were further incubated for 14 days. Colonies were almost completely absent at 10 µM in the MDA-MB-231 cells and 5 µM in Hs578T cells (Figure 1D,E). The IC_50_ calculated from the clonogenic assay results in the MDA-MB-231 and Hs578T cells were 0.92 µM and 0.58 µM, respectively. Similar to the SRB results, FDI-6 significantly suppressed the long-lasting clonogenic potential of TNBC in a dose-dependent manners (Figure 1F,G). 

**Figure 2:**
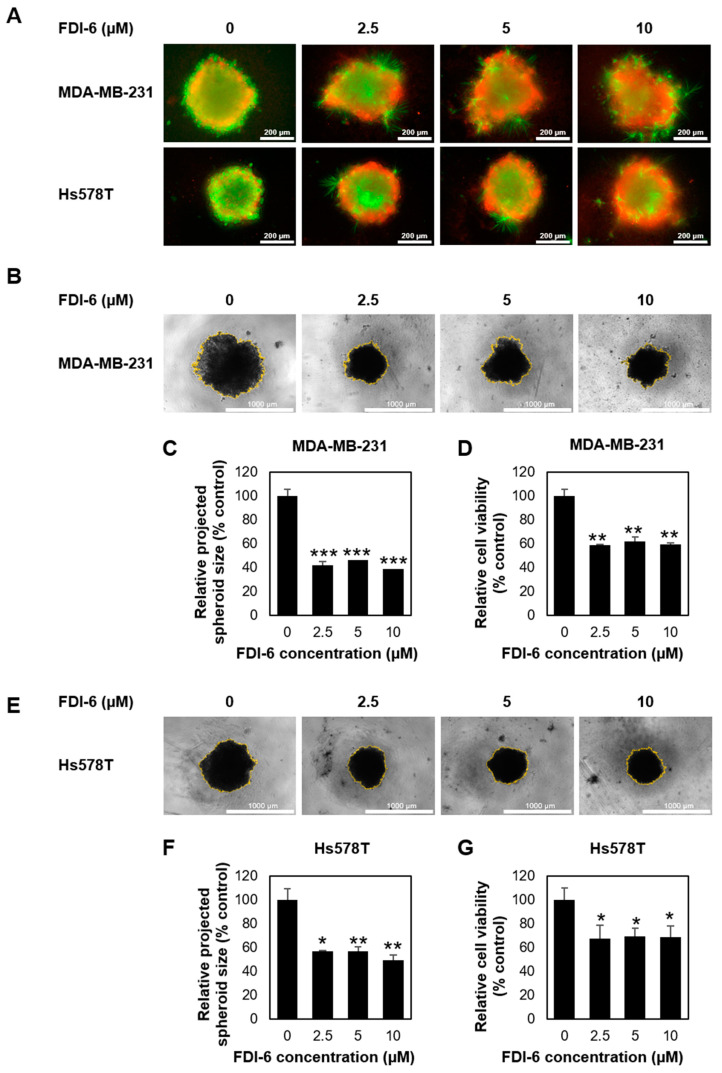
Effect of FDI-6 on the viability of the TNBC cells in 3D spheroid assays. (**A**) Live/dead staining (live = green and red = dead) of the MDA-MB-231 and Hs578T spheroids after being treated with different concentrations of FDI-6 for 72 h; (**B**) Representative images of the MDA-MB-231 spheroid size analysis after being treated with different concentrations of FDI-6 for 72 h and being incubated for a total of 21 days; (**C**) Relative size and (**D**) relative cell viability of FDI-6 treated MDA-MB-231 spheroids in comparison with the untreated spheroid; (**E**) Representative images of the Hs578T spheroid size analysis; (**F**) Relative size and (**G**) relative cell viability of the FDI-6 treated Hs578T spheroids in comparison with the untreated spheroid. All bar graphs represent an average of three independent experiments ± S.D. (*n* = 3). Statistical significance was determined by Student’s *t*-test (significant, * *p* < 0.05, ** *p* < 0.01, *** *p* < 0.001).

Next, we performed a 3D spheroid formation assay, a more physiological approach for evaluating tumour growth. Spheroids generated on ultra-low attachment plates were treated with or without FDI-6 at various concentrations for 72 h and were incubated further for 14 days. Spheroid’s viability was firstly visualized using a live/dead cell staining kit (Thermo Fisher Scientific, Waltham, MA, USA) after 72 h. As seen in Figure 2A, an obviously higher number of dead cells was observed in all FDI-6 treated spheroids, possibly due to the effective diffusion capacity of the compound into the tumour spheroids, as well as increased levels of cellular stress inside the 3D microstructure compared with that of the untreated spheroids. The projected area spheroid images were then used as a measurement of the spheroid size to evaluate the anti-tumour growth activity at day 21 (Figure 2B,E). A reduction in the projected area of the spheroids was quantified as a percentage change of FDI-6 treated spheroids compared with the untreated control spheroids. The results showed that both MDA-MB-231 and Hs578T spheroids, which were treated with FDI-6, even at a low concentration such as 2.5 µM, were considerably smaller than the untreated spheroids from the same cell lines (Figure 2C,F). Additionally, the anti-proliferative effect on the 3D spheroid model was also quantitively measured using a 3D CellTiter-Glo luminescent cell viability assay (Promega, Madison, WI, USA). Similar to the results from the size measurement, the viability of the treated spheroids was significantly reduced compared with the untreated spheroids (Figure 2D,G). Taken together, these results strongly suggested that FDI-6 effectively inhibits TNBC cell growth.

### 2.2. FDI-6 Inhibits TNBC Cell Migration and Invasion

**Figure 3:**
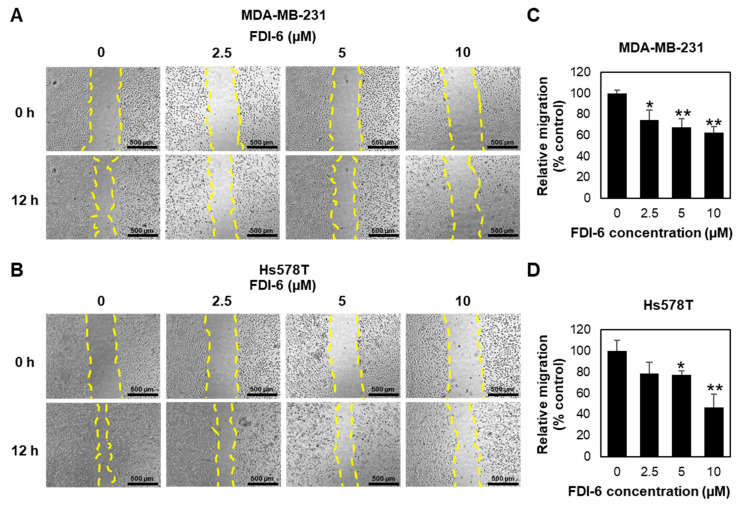
FDI-6 inhibited the migration of breast cancer cells. (**A**,**B**) Representative images of the wound healing assay of (**A**) MDA-MB-231 and (**B**) Hs578T cells after 12 h of FDI-6 treatment at indicated concentrations; (**C**,**D**) The bar graphs represent the average of relative migration of (**C**) MDA-MB-231 cells and (**D**) Hs578T cells in comparison with the control from three independent experiments ± S.D. (*n* = 3). Statistical significance was determined by Student’s *t*-test (significant, * *p* < 0.05 and ** *p* < 0.01).

In addition to the anti-proliferative activity, we evaluated the anti-migratory activity of FDI-6 by performing a wound healing assay on MDA-MB-231 and Hs578T cells (Figure 3A,B). Mitomycin C was used for 2 h to inhibit cell proliferation. The wounds were then generated by scratching the monolayer of cells, and the cells were then treated with various concentrations of FDI-6 (0, 2.5, 5, or 10 µM) for 12 h. As shown in Figure 3A,B, FDI-6 significantly suppressed the ability of the TNBC cells, as well as both MDA-MB-231 and Hs578T cells, to migrate and fill up the wound area. The quantitatively relative changes in the wound area, as presented in Figure 3C,D, indicated that FDI-6 suppressed the migration ability of TNBC cells in a dose dependent manner. 

**Figure 4:**
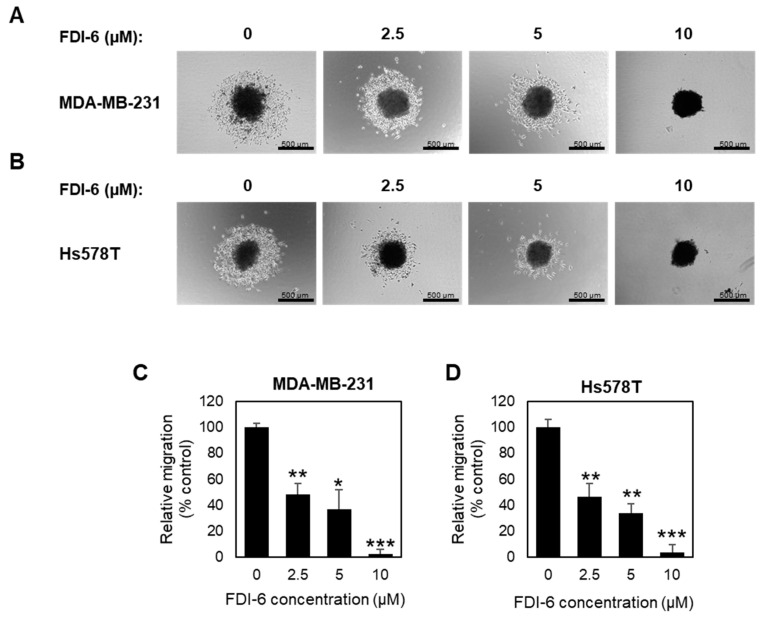
Effect of FDI-6 on the migration of TNBC cells in 3D spheroid assays. (**A**,**B**) Representative images of (**A**) MDA-MB-231 and (**B**) Hs578T spheroids after being treated with 0 µM, 2.5 µM, 5 µM, and 10 µM of FDI-6 for 72 h, after being transferred to a flat bottom culture plate and further incubated for 24 h. Scale bar = 500 µm; (**C**,**D**) The bar graphs represent the average of the relative migration of (**C**) MDA-MB-231 cells and (**D**) Hs578T cells in comparison with the control from three independent experiments ± S.D. (*n* = 3). Statistical significance was determined by Student’s *t*-test (significant; * *p* < 0.05, ** *p* < 0.01, *** *p* < 0.001).

Next, we also confirmed the anti-migrative effect using a 3D migration assay. Spheroids were subjected to pre-treat with mitomycin C for 2 h, and followed with FDI-6 treatment for 72 h, and were then transferred to flat-bottom plates. After 24 h from the transfer, the migration areas were measured and calculated relatively to the spheroid areas (Figure 4A,B). Similar to the wound healing assay results, the migration activities were significantly reduced in the treated spheroids (Figure 4C,D), confirming that FDI-6 significantly suppressed TNBC cell migration. 

**Figure 5:**
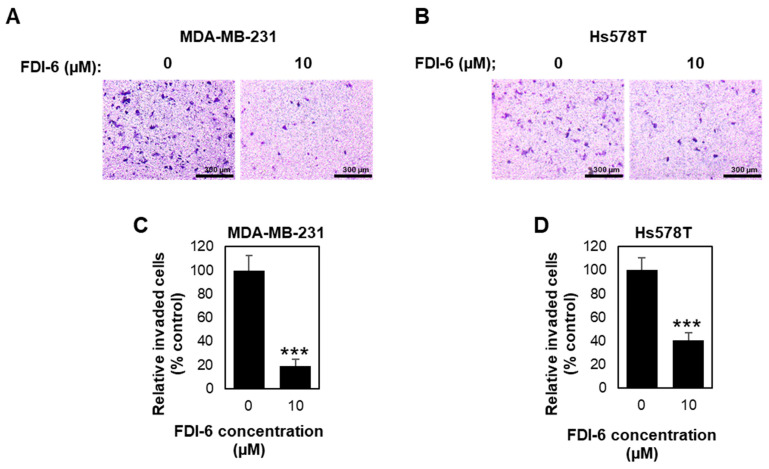
FDI-6 suppressed the invasion of TNBC cells. (**A**,**B**) Representative image from the Transwell invasion assay of the (**A**) MDA-MB-231 and (**B**) Hs578T cells after being treated with 0 µM and 10 µM FDI-6 for 24 h; (**C**,**D**) The bar graphs represent the average relative invasion of (**C**) MDA-MB-231 and (**D**) Hs578T cells in comparison with the control from three independent experiments ± S.D. (*n* = 3). Statistical significance was determined by Student’s *t*-test (significant, *** *p* < 0.001).

We then examined the FDI-6 effect on cell invasion by performing a transwell invasion assay. Briefly, TNBC cells were pre-treated with mitomycin C for 2 h to inhibit cell proliferation, followed with FDI-6 treatment for 24 h before seeding into the transwell chamber, and the cells invaded through the Matrigel layer were stained and calculated (Figure 5A,B). We found that treatment with FDI-6 significantly reduced the number of invaded cells in comparison with the control condition (Figure 5C,D), indicating that FDI-6 suppressed the invasion ability of the TNBC cells. Thus, our results suggest that FDI-6 could lead to the significant inhibition of the migration and invasion of TNBC cells.

### 2.3. FDI-6 Suppressed Gene Expression Related to Tumour Progression of TNBC Cells

**Figure 6:**
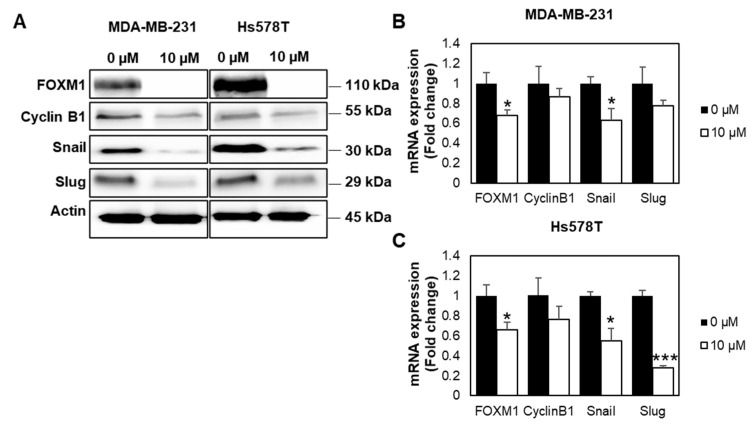
FDI-6 suppressed gene expression related to cancer progression. (**A**) Image from Western blot analysis of the HS578T and MDA-MB-231 cells after being treated with 0 and 10 µM FDI-6; (**B**,**C**) The relative mRNA expression levels of FOXM1 and its oncogenic targets of the treated (**B**) MDA-MB-231 and (**C**) Hs578T cells in comparison with the control determined by qRT-PCR and were normalized against L19, the house keeping gene. The bar graphs represent the average of three independent experiments ± S.D. (*n* = 3). Statistical significance was determined by Student’s *t*-test (significant, * *p* < 0.05 and *** *p* < 0.001).

Next, we investigated the molecular mechanisms related to cancer progression using both Western blot analysis and qPCR. From the Western blot analysis (Figure 6A, Figure S3), we found that FOXM1 was downregulated in the FDI-6 treated cells. We also investigated the epithelial–mesenchymal transition (EMT) process, which is an important aspect in invasion and metastasis, allowing the cells to detach from their primary site and move through the basement membrane [19]. Interestingly, the protein expression levels of Snail and Slug, key factors of the EMT process, were also significantly reduced in the FDI-6 treated cells. Consistently, the qPCR analysis of the MDA-MB-231 (Figure 6B) and Hs578T cells (Figure 6C) treated with 10 µM of FDI-6 exhibited a significant reduction of FOXM1 mRNA expression. Cyclin B1, which is a FOXM1 downstream target, related to cell cycle progression and proliferation, was decreased [20,21,22]. The Snail mRNA expression levels were also shown to be decreased. Overall, the results suggest that FDI-6 suppressed the transcriptional expression of FOXM1, and its downstream targets are related to the proliferation, invasion, and metastasis of TNBC cells.

### 2.4. FDI-6 Promotes Caspase-3 Activation, Cleavage of PARP, and Bcl-2 Expression

**Figure 7:**
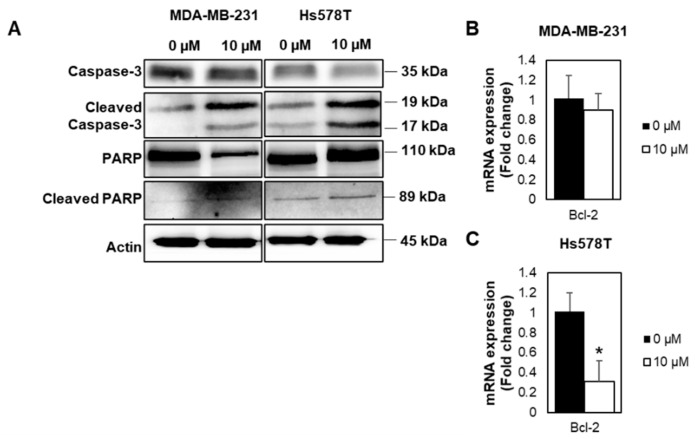
FDI-6 promotes Caspase-3 activation, cleavage of PARP, and Bcl-2 expression. (**A**) Image from the Western blot analysis of HS578T and MDA-MB-231 cells after being treated with 0 and 10 µM FDI-6; (**B**,**C**) The relative Bcl-2 mRNA expression level of the treated (**B**) MDA-MB-231 and (**C**) Hs578T cells in comparison with the control were determined by qRT-PCR and were normalized against L19, the house keeping gene. The bar graphs represent the average of three independent experiments ± S.D. (*n* = 3). Statistical significance was determined by Student’s *t*-test (not significant, ns; significant, * *p* < 0.05).

FOXM1 depletion has been reported to inhibit cancer cell growth and trigger apoptosis [23,24]. We thus investigated if the growth inhibition following FDI-6 treatment is related to the induction of apoptosis. The results show that the cleavage of PARP and Caspase-3, known markers of apoptosis, were increased in the FDI-6 treated cells (Figure 7A, Figure S3). Consistently, the transcriptional expression of Bcl-2, known as a cell survival protein, which inhibits apoptosis, was decreased in the FDI-6 treated cells (Figure 7B,C). These results suggest the induction of apoptosis cell death in FDI-6 treated cells.

### 2.5. Synergistic Effect of FDI-6 in Combination with Doxorubicin against TNBC Cells

**Figure 8:**
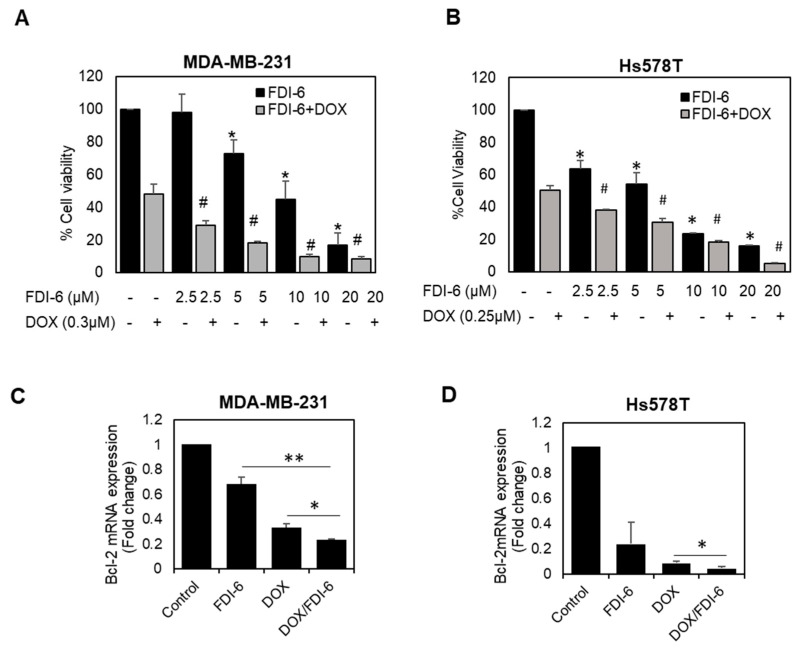
Synergistic effect of the combination of FDI-6 and doxorubicin. (**A**,**B**) The cytotoxic effects using SRB assay of (**A**) MDA-MB-231 and (**B**) Hs578T cells treated with different combination of FDI-6 and doxorubicin; (**C**,**D**) The relative Bcl-2 mRNA expression level of the treated (**C**) MDA-MB-231 and (**D**) Hs578T cells with an IC50 concentration of DOX, FDI-6, and DOX/FDI-6 combination. The bar graphs represent the average of three independent experiments ± S.D. (*n* = 3). Data analysis was performed using one-way ANOVA. A *p*-value < 0.05 was considered significantly different and denoted as follows: * *p* < 0.05 and ** *p* < 0.01 representing significance compared with control, # *p* < 0.05 representing significance compared with DOX alone.

Doxorubicin-based chemotherapy is currently the most frequently used treatment for triple negative breast cancer (TNBC); however, its therapeutic outcomes are limited by its toxicity and chemoresistance. FOXM1 has been reported to play a vital role in chemotherapy resistance [11,25]. Combination therapy using chemotherapeutic agents together with targeted therapy has currently become a potential strategy for cancer management, expected to improve the therapeutic efficacy and decrease side effects. In this study, we also investigated the potency of FDI-6 when used in combination with doxorubicin. The results show that the IC_50_ concentration of DOX with FDI-6 in combination significantly suppressed cell proliferation and increased the apoptotic cell death of TNBC cells, as indicated by the SRB results (Figure 8A,B), and the down-regulation of the anti-apoptotic protein expression, BCL-2 (Figure 8C,D), suggesting that FDI-6 could effectively sensitize TNBC to doxorubicin in a dose-dependent manner. To confirm and quantify this synergistic effect further, the combination index (CI) was then computed for the combination of FDI-6/DOX in both MDA-MB-231 and Hs578T using CompuSyn software. As shown in Table 1, a CI range from 0.58 to 0.99, corresponding to the fraction affected (Fa) values from 0.66 to 0.95, further confirmed a synergism between the two drugs for inhibiting the proliferation of TNBC cells. 

## 3. Discussion

Triple negative breast cancer (TNBC) is considered to be a clinical challenge due to its aggressive nature and the absence of specific molecular targets for treatment. Interestingly, previous studies have demonstrated that FOXM1 is upregulated in TNBC [12,13,14]. Therefore, targeting FOXM1 could be a potential strategy for treating TNBC. FDI-6 has been identified as a recent FOXM1 inhibitor [16]. However, its anti-cancer effects in TNBC cells are not clearly understood. In this study, we therefore evaluated the anti-cancer effects, including the anti-proliferative as well as anti-metastatic activity, of FDI-6 in TNBC cells.

Using both 2D monolayer cultures and 3D spheroid assays, our results demonstrated for the first time that FDI-6 significantly suppressed the cell proliferation of TNBC cells in both 2D monolayer cultures and 3D spheroid assays. The effect was possibly related to the inhibition of FOXM1. This was confirmed by the findings from qPCR and Western blot analysis, which demonstrated the downregulation of FOXM1 and its key cell cycle related downstream target, Cyclin B1, at both mRNA and protein levels, after treating the cells with FDI-6 for 24 h. Several studies have previously reported that FOXM1 is a crucial regulator of cell cycle progression. Its expression is significantly increased during mitosis, reaching its peak during the G2/M phase. Cyclin B1 has also been identified as a key essential target for mitotic entry [21,26]. Consistently, the specific inhibition of FOXM1 using different strategies, such as siRNA [27], thiostrepton [28], or peptide inhibitor [29], has previously been reported to significantly suppress tumour growth in various in vitro and animal models. The anti-proliferative effect has also been reported in other cell types, including, human laryngeal carcinoma Hep-2 cells [30] and primary multiple myeloma cells [31]. However, there was a notable difference in results between our study and previous studies by Gormally et al. and Liu et al. Both of these studies reported that only the nuclear FOXM1 protein level was decreased, while the total FOXM1 remained unaffected [16,30]. However, our results demonstrated the total FOXM1 downregulation after a longer treatment duration of 24 h. This is possibly due to the positive autoregulatory loop of FOXM1, which activated its own transcription activity, increasing its mRNA expression and protein. Therefore, inhibiting the FOXM1 transcription activity by FDI-6 affected the autoregulatory loop and caused a further reduction in FOXM1 expression in both the mRNA and protein levels [32]. In addition, Ziegler et al. recently reported that the new class of compounds (NB-73), which exhibited a similar function to FDI-6, binds directly to FOXM1 and alters its proteolytic sensitivity, reducing the cellular level of the FOXM1 protein through a proteasome-dependent process. Therefore, the reduction of FOXM1 expression after treatment with FDI-6 might be also related to the proteolytic process [33]. The proteasome inhibitor MG132 treatment experiment needs to be performed to further confirm this.

Additionally, we also observed that FDI-6 at a low concentration such as 2.5 µM can remarkably inhibit TNBC cell proliferation in long term assays. The low dose therapeutic effect could be utilised in patients with a low tolerance to the side effects of the treatment. The usage of prolonged low doses as an alternative to the usual doses has already been studied in other therapeutic agents, known as low-dose metronomic (LDM) chemotherapy. Several clinical trials have demonstrated the benefits in the clinical safety of LDM chemotherapy without any significance reduction in the anti-cancer effects of chemotherapeutic agents [34]. 

In addition to the anti-proliferative effect, we also investigated further and found that FDI-6 induced apoptosis in TNBC cells by activating apoptosis related proteins and PARP cleavage. Previous studies have reported that knocking down FOXM1 sensitized cancer cells to apoptosis [24] and also improved the efficiency of other treatments, such as irradiation or chemotherapeutic agents [10,11]. Other FOXM1 inhibitors, such as Siomycin A [35] and thiostrepton [36], have been reported to induced apoptosis in cancer cells. In addition, inhibiting FOXM1 has been shown to induce apoptosis in several cancer cell types, including breast cancer [37], ovarian cancer [38], and nasopharyngeal carcinoma [39].

Moreover, we observed FDI-6 inhibited TNBC cell growth in a FOXM1 expression-dependent manner. As shown in the anti-proliferative assays, TNBC cells, which exhibited a higher FOXM1 expression, were effectively suppressed by FDI-6 at considerably low concentrations compared with the non-TNBC cells, which had a lower FOXM1 expression level. These findings suggest that FDI-6 had a specificity toward high FOXM1 expressing cell types. This result was also strongly supported by previous molecular docking studies, which demonstrated that FDI-6 bind directly to the DNA binding domain of FOXM1 by utilising the specific π–sulphur interaction (between a His287 and a sulphur containing heterocycle) [40] and halogen-bonding interaction (between Arg297 and halogen group (4-fluorophenyl) in FDI-6 structure) [41]. These specific binding interactions between FDI-6 and the FOXM1 DNA binding domain have been suggested to contribute to the specificity of FDI-6 toward FOXM1.

Next, the inhibitory effects of FDI-6 on cell migration and invasion were also investigated. Our findings show that FDI-6 notably suppressed cell migration and invasion. Regarding these aspects, several studies have reported the positive correlation between FOXM1 expression and cancer cell migration and invasion [42,43], as well as inhibiting FOXM1 via different methods, which resulted in a reduction in these activities of cancer cells [44,45]. Furthermore, another two identified FOXM1 downstream targets, Snail [46] and Slug [47], were found to be downregulated in FDI-6 treated cells in the present study. These two proteins are the main transcriptional factors that are involved in the EMT processes [48]. This suggests that FDI-6 also inhibited EMT in cancer progression. In line with our results, previous studies have reported that the suppression of FOXM1 [49], Snail [49,50], or Slug [51] via the RNA interference would result in the inhibition of EMT progression. Another noteworthy finding is that E-cadherin was upregulated in the treated Hs578T cells (Figure S2). It is an adhesion protein that is repressed by Slug and Snail during the EMT process [48,52]. Loss of E-cadherin further contributed to the cancer progression by inducing multiple changes in the transcriptional and functional activities, leading to metastatic dissemination [52]. This finding further supports the inhibitory effect of FDI-6 on EMT and cancer metastasis. Taken together, the data suggest that FDI-6 could suppress migration and invasion, especially in the EMT process, via the inhibition of FOXM1 and the downregulation of Snail and Slug. 

Interestingly, we also investigated a synergistic effect of the combination of FDI-6 and doxorubicin, and found that the FDI-6/DOX combination significantly enhanced the cytotoxicity and apoptotic properties, suggesting that FDI-6 might improve doxorubicin treatment efficacy and decrease the required dose, thus reducing the unwanted side effects. In addition, FOXM1 plays a vital role in chemotherapy resistance; therefore, targeting FOXM1 using FDI-6 might also prevent the occurrence of doxorubicin resistance and re-sensitize the resistant cells to doxorubicin. Consistently, similar synergistic effects of the combination of specific FOXM1 inhibition strategies, such as siRNA, shRNA, inhibitor, and aptamers, against FOXM1, together with doxorubicin, have been previously reported to sensitize various human cancer cells to apoptosis induced by DNA-damaging agents, including doxorubicin [11,25,53,54]. 

In conclusion, our findings demonstrated that FDI-6 suppressed cancer cell proliferation, migration, and invasion via the downregulation of FOXM1 and its downstream targets. In addition, we also demonstrated that FDI-6 specifically targeted cancer cells with a high FOXM1 expression, which can be very important for cancer treatment. Considering these findings, we suggest that FDI-6 could be a promising candidate for TNBC treatment. However, further in vitro studies in other cells with an overexpression of FOXM1 and in vivo studies are required to confirm its activities. Additionally, the development of a drug delivery system for FDI-6 should also be considered so as to drive the effective therapeutic window to achieve clinical benefits in TNBC treatment.

## 4. Materials and Methods 

### 4.1. Cell Culture

All cell lines originated from the American Type Culture Collection (Manassas, VA, USA). Cells were cultured in Dulbecco modified Eagle medium (DMEM) supplemented with 10% fetal bovine serum (FBS), GlutaMAX, and Penicillin-Streptomycin (10,000 U/mL). DMEM and all of the supplements were purchased from Gibco (Life Technologies Ltd., Paisley, UK). The cells were maintained at 37 °C in a humidified incubator with 5% CO_2_.

### 4.2. 3D Spheroid Formation

Spheroids were formed using an ultra-low attachment (ULA) 96-well plate (Corning, Kennebunk, ME, USA). The cells were seeded as 5 × 10^3^ cells/well in 100 μL of 10% FBS DMEM, and were then incubated at 37 °C for 3 days in a humidified incubator with 5% CO_2_.

### 4.3. Sulforhodamine B (SRB) Assay

The cells were seeded in 96-well culture plates as 5000 cells/well in 100 µL completed DMEM media, and were incubated at 37 °C overnight before adding FDI-6 (SML1392, Sigma Aldrich, Singapore) in varied concentrations. After being incubated further for 3 days, the cells were washed with PBS, fixed with 10% trichloroacetic acid for 1 h, and then washed again with PBS. Then, 1% SRB solution was added to the 50 µL/wells for staining. The samples were incubated for 30 min at room temperature and rinsed three times with 1% acetic acid afterward. The culture plates were dried at room temperature overnight. The stained samples were dissolved in 50 µL of 10 mM Tris base solution and the absorbance was measured at 560 nm. The inhibitory concentration 50% (IC_50_) was determined by using the IC_50_ calculator tool of the AAT Bioquest webpage [55].

### 4.4. Clonogenic Assay

The cells were seeded in 24-well culture plates as 250 cells/well with 500 μL of DMEM supplemented with 10% FBS added into the wells. After being incubated at 37 °C overnight to let the seeded cells attach to the culture plates, the media were replaced with the one that contained a variable concentration of FDI-6. The cells were then incubated for 72 h before switching back to normal media without FDI-6, and continued to be incubated for a total of 14 days. Once the appropriate amount of cell colonies formed, they were fixed with 4% paraformaldehyde for 20 min, stained with crystal violet solution for 1 h, and then washed under running tap water. The culture plates were dried at room temperature overnight. Finally, the stained colonies were dissolved in 10% acetic acid and the absorbance was measured at 590 nm using microplate readers (Thermo Fisher Scientific, MA, USA). The cell viability data, derived from the reader, were calculated into the relative percentage with the control group. The inhibitory concentration 50% (IC_50_) was determined using the IC_50_ calculator tool of the AAT Bioquest webpage [55].

### 4.5. Wound Healing Assay

The cells were seeded in 24-well culture plates as 1 × 10^5^ cells/well in DMEM supplemented with 10% FBS, and were incubated at 37 °C until confluent cell monolayers were formed. The cells were then pre-treated with mitomycin C (Ametycin 10 µg/mL; Tokyo Chemical Industry Co., Ltd. (TCI)), Tokyo, Japan) for 2 h at 37 °C and 5% CO_2_ to inhibit cell proliferation. After that, the cell monolayers were scratched with 200 μL pipette tips to form gap wounds. The media in the wells were replaced with DMEM supplemented with 1% FBS and added FDI-6 in varied concentrations. The cells were incubated at 37 °C and the images of the wounds were captured under a microscope periodically using an automated live cell imager, LionheartFX (Biotek, Winooski, VT, USA). Finally, the gap distances in the captured images were measured using Gen5 software (Biotek, VT, USA). The measured data were calculated into the relative percentage of the control group.

### 4.6. Invasion Assay

Invasion assays were done in transwell invasion chambers with an 8 μm pore size filter membrane (Corning, ME, USA). The cells were treated with FDI-6 before the experiment 24 h prior. The inserts were coated with 100 µL of 0.3 mg/mL of Matrigel (Corning, Kennebunk, ME, USA) and were incubated at 37 °C overnight. After the Matrigel layer formed inside the inserts, the cells were pre-treated with mitomycin C (10 µg/mL) for 2 h and were then transferred into each insert as 6.5 × 10^4^ cells/well in 200 μL of DMEM supplemented with 1% FBS. The lower chambers were added with 600 μL of DMEM supplemented with 10% FBS. The inserts were then merged with the lower chambers and were incubated at 37 °C for 16 h. After incubation, the cells that did not migrate through the pores were removed with cotton swabs. The cells on the underside of the inserts were fixed with 25% (*v*/*v*) methanol for 15 min and then stained with 1% (*w*/*v*) crystal violet in 25% (*v*/*v*) methanol for 15 min. The stained cells were washed with distilled water for 30 s. The inserts were dried at room temperature overnight. The images of the stained cells on the underside were obtained by an inverted microscope (Olympus, Tokyo, Japan). The average number of migrated cells were calculated from five randomly chosen fields from each insert.

### 4.7. Live/Dead Staining

Live/dead viability/cytotoxicity kits (Thermo Fisher Scientific, Waltham, MA, USA) were used for staining. The solution contained calcein-AM, which interacted with live cells, and ethidium homodimer-1 for the dead cells. The cells were cultured in 96-well plates and additional media were removed until 100 µL were left in each well. Then, 30 µL of the mixed solution was added to every well. The plates were wrapped with aluminium foil to avoid direct contact with sunlight and were then incubated at 37 °C for 30 min. The images were obtained with a LionheartFX live cell imager (Biotek, Winooski, VT, USA).

### 4.8. 3D Proliferation Assay

The prepared spheroids were treated with various concentrations of FDI-6. After that, the media were removed until 100 µL remained in each well. Then, 100 µL of CellTiter-Glo 3D cell viability assay reagent (Promega, Madison, WI, USA) was added and incubated for another 30 min. The contents in the wells were then mixed thoroughly for 5 min and were incubated at room temperature for 25 min. Luminescence data were obtained using the luminometer function of a microplate reader (Thermo Fisher Scientific, MA, USA). Data were calculated relative to the control group.

### 4.9. 3D Migration Assay

The cells were prepared as 3D spheroids in ULA plates, as previously mentioned. After treatment, the cells were pre-treated with mitomycin C (10 µg/mL) for 2 h and then the spheroids were transferred to the flat bottom microplates, thus allowing the cells to attach to the bottom. The cells were incubated for another 24 h. Images of the transferred spheroids were obtained using a LionheartFX live cell imager (Biotek, Winooski, VT, USA). The migration area was measured from the images using ImageJ software [56] and was calculated relatively to the spheroid area.

### 4.10. Western Blotting

The cell lysates were prepared using a RIPA buffer (Thermo Scientific, St. Peters, MO, USA) with a protease inhibitors mixture (Roche Applied Science, Mannheim, Germany). Lysate was agitated for 30 min at 4 °C then centrifuged at 14,000 rpm for 10 min at 4 °C to collect the supernatants, and then the protein concentration was measured using the Bio-Rad DC Protein Assay (Biorad, UK). Then, 20 µg of each protein sample was then loaded onto 4–12% Tris-Glycine mini gels (Invitrogen), and was transferred to nitrocellulose membrane (GE Healthcare, Amersham, Buckinghamshire, UK). The membranes were incubated with the indicated antibodies, and the FOXM1 (C-20) (Cat#sc-502) antibody was purchased from Santa Cruz Biotechnology (Santa Cruz Biotechnology, Santa Cruz, CA, USA). Other antibodies were purchased from Cell Signalling Technology (New England Biolabs Ltd., Hitchin, UK). Primary antibodies were detected using horseradish peroxidase-linked anti-mouse or anti-rabbit conjugates, as appropriate (Dako, Glostrup, Denmark), and were visualized using the UVITEC chemiluminescence imaging platform (UVITEC, UK). The intensities of the protein bands were then quantitated using an ImageJ program and were normalized by dividing the intensity of the bands with the that of the actin band as the control.

### 4.11. Real-Time Quantitative PCR (qPCR) Assay

The total RNA extraction was done using an RNeasy Mini Kit (Qiagen, Hilden, Germany), according to the manufacturer’s instructions. Then, 1 µg of the total cellular RNA for each of the conditions were used to generate complementary DNA using Superscript III reverse transcriptase and oligo-dT primers (Invitrogen, Paisley, UK). qPCR was done using HOT FIREPol EvaGreen qPCR Mix Plus (no ROX). The amplification was performed on a CFX Connect real-time system (Bio-Rad, Hercules, CA, USA). The results were normalized using the L19 mRNA expression as a reference gene. All of the qPCR experiments were repeated at for least three independent experiments, and the relative expression shown as average ± S.D. (*n* = 3). Statistical significance was determined by Student’s *t*-test (significant, * *p* < 0.05, ** *p* < 0.01, *** *p* < 0.001). Primer sequences are described in Table 2.

### 4.12. Quantitative Determination of Drug Synergism 

Combination index (CI) values were calculated to determine whether FDI-6 synergistically enhanced the cytotoxicity of DOX. The association between the effects of FDI-6 and DOX alone and in combination were analyzed using CompuSyn 1.0 software (Combosyn Inc., Paramus, NJ, USA), as previously described [57]. The CI values were determined for each dose and the corresponding effect level was presented as the fraction affected (Fa). The CI values obtain a quantitative definition for the synergism (CI < 1), additive effect (CI = 1), and antagonism (CI > 1) of the FDI-6/DOX combinations.

### 4.13. Statistical Analysis

All of the results of both functional studies, qPCR, and the Western blot analysis were performed in at least three independent experiments ± S.D. (*n* = 3). Statistical significance was determined by Student’s *t*-test compared with the control/untreated conditions (significant, * *p* < 0.05, ** *p* < 0.01, *** *p* < 0.001) using Windows 10, Excel. For drug synergism studies, data analysis was performed using one-way ANOVA, and a *p*-value < 0.05 was considered significantly different.

## Figures and Tables

**Table 1:** CI analysis of FDI-6 combined with doxorubicin used to treat TNBC cells using CompuSyn software.

Cell Line	[FDI-6] (µM)	[Doxorubicin] (µM)	Fa	CI
MDA-MB-231	2.5	0.3	0.71	0.74
	5	0.3	0.82	0.62
	10	0.3	0.90	0.58
	20	0.3	0.92	0.84
Hs578T	2.5	0.25	0.66	0.99
	5	0.25	0.75	0.98
	10	0.25	0.84	0.97
	20	0.25	0.95	0.58

The CI values obtained a quantitative definition for the synergism (CI < 1), additive effect (CI = 1), and antagonism (CI > 1).

**Table 2:** **List of** primer sequences used in this study.

Gene	Forward Primer (5′ > 3′)	Reverse Primer (5′ > 3′)
FOXM1	TGCAGCTAGGGATGTGAATCTTC	GGAGCCCAGTCCATCAGAACT
Cyclin B1	AAGAACAGCTCTTGGGGACA	CACTGGCACCAGCATAGGTA
Snail	GGTTCTTCTGCGCTACTGCT	TAGGGCTGCTGGAAGGTAAA
Slug	GCCAAACTACAGCGAACTGG	GATGGGGCTGTATGCTCCTG
Bcl-2	CTTTGAGTTCGGTGGGGTCA	GGGCCGTACAGTTCCACAAA
L19	GCGGAAGGGTACAGCCAAT	GCAGCCGGCGCAAA

## Supplementary Materials

The following are available online at https://www.mdpi.com/article/10.3390/ijms22136685/s1.

## Abbreviations

Bcl-2	B-cell lymphoma 2
DMEM	Dulbecco modified Eagle medium
ER	Estrogen receptor
FBS	Fetal bovine serum
FOXM1	Forkhead box protein M1
G2/M	Gap 2/Mitosis
IC_50_	Half maximal inhibitory concentration
L19	60S ribosomal protein L19
PBS	Phosphate-buffered saline
PR	Progesterone receptor
qPCR	Quantitative polymerase chain reaction
siRNA	Small interfering RNA
Snail	Zinc finger protein SNAI1
SRB	Sulforhodamine B
TNBC	Triple negative breast cancer
ULA	Ultra-low attachment
