# Supplementary material for: The Anticancer Effects of FDI-6, a FOXM1 Inhibitor, on Triple Negative Breast Cancer
Source: Int J Mol Sci. 2021 Jun 22;22(13):6685. doi: 10.3390/ijms22136685 (PMC8269391; doi:10.3390/ijms22136685)
Supplement: Supplementary file 1 [file ijms-22-06685-s001.zip › ijms-1236162-supplementary.pdf]

Supplementary Figure 1

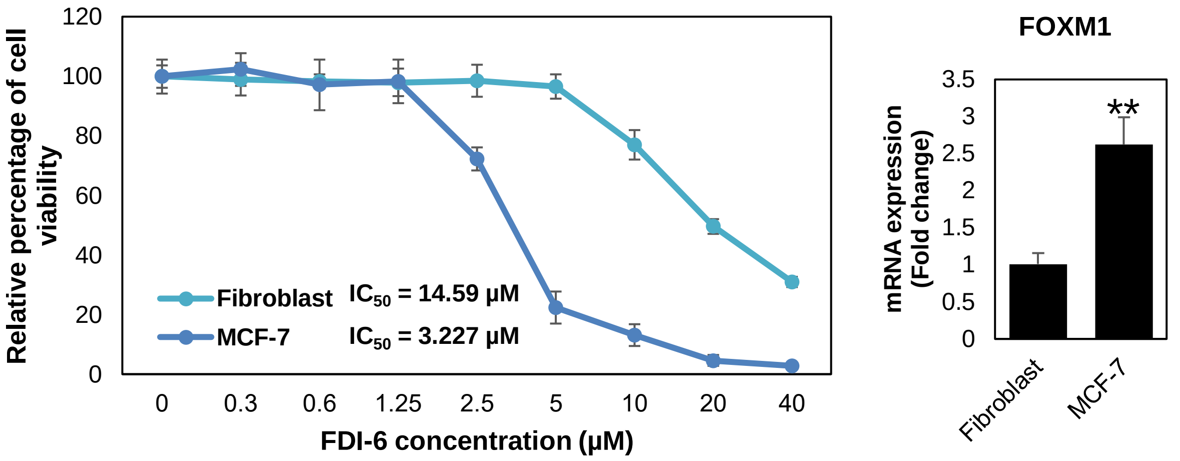

Figure S1: Anti-proliferative effects on Fibroblasts and ER-positive MCF7 cells (A) SRB assay results (B) mRNA expression level of FOXM1 in the cell lines used in this study determined by qRT-PCR after normalizing against L19 the house keeping gene. Graphs represent an average of three independent experiments ± S.D. (n = 3). Statistical significance was determined by Student's t-test (Significant; ** $p < 0.01$).

Supplementary Figure 2

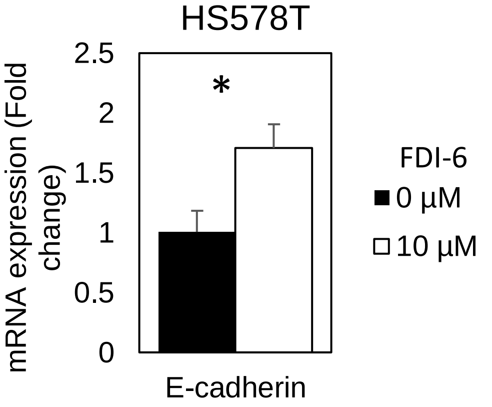

Figure S2: FDI-6 significantly increased E-cadherin mRNA expression in cells Hs578T cells. mRNA expression level of E-cadherin in this study determined by qRT-PCR after normalizing against L19 the house keeping gene. Graph represent an average of three independent experiments± S.D. (n = 3). Statistical significance was determined by Student's t-test (Significant; $*p < 0.05$).

Supplementary Figure 3

A)

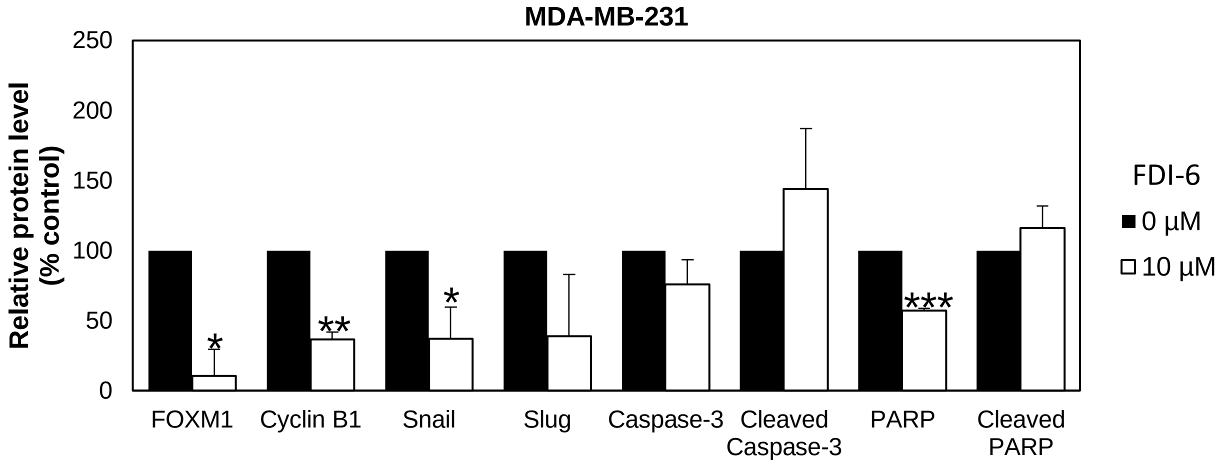

B)

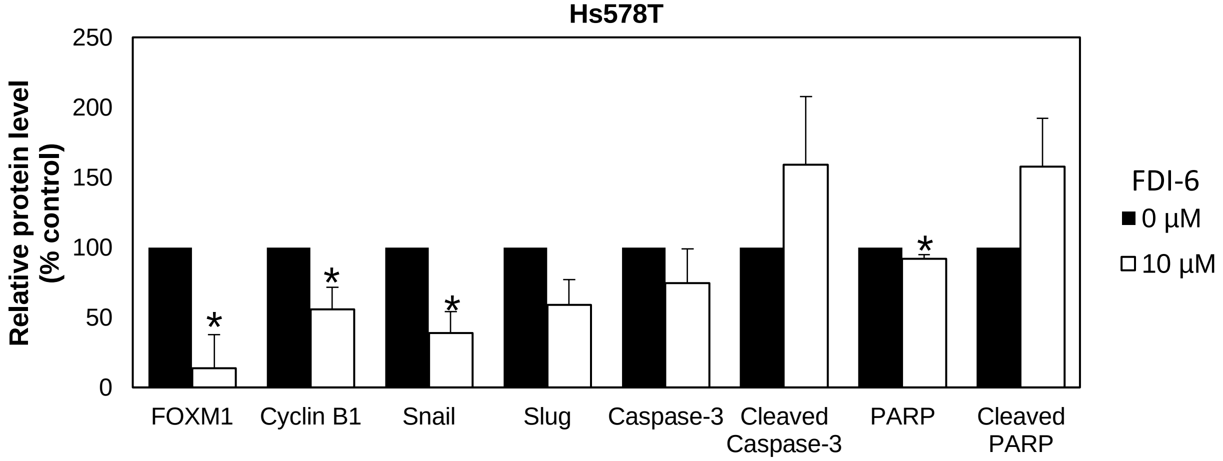

Figure S3. Quantification of western blot analysis, related to Figure 6 and Figure 7. The intensities of protein bands were quantitated using ImageJ program and normalized by dividing the intensity of the bands with the that of actin band, as control. Graph represent an average of three independent experiments ± S.D. (n = 3). Statistical significance was determined by Student's t-test (Significant; * $p < 0.05$, ** $p < 0.01$, *** $p < 0.001$).
